# Synthesis and In Vitro Inhibition Effect of New Pyrido[2,3-d]pyrimidine Derivatives on Erythrocyte Carbonic Anhydrase I and II

**DOI:** 10.1155/2014/594879

**Published:** 2014-08-04

**Authors:** Hilal Kuday, Fatih Sonmez, Cigdem Bilen, Emre Yavuz, Nahit Gençer, Mustafa Kucukislamoglu

**Affiliations:** ^1^Department of Chemistry, Faculty of Art and Sciences, Sakarya University, 54140 Sakarya, Turkey; ^2^Pamukova Vocational High School, Sakarya University, 54900 Sakarya, Turkey; ^3^Department of Chemistry, Faculty of Art and Sciences, Balikesir University, 10145 Balikesir, Turkey

## Abstract

In vitro inhibition effects of indolylchalcones and new pyrido[2,3-d]pyrimidine derivatives on purified human carbonic anhydrase I and II (hCA I and II) were investigated by using CO_2_ as a substrate. The results showed that all compounds inhibited the hCA I and hCA II enzyme activities. Among all the synthesized compounds, **7e** (IC_50_ = 6.79 *µ*M) was found to be the most active compound for hCA I inhibitory activity and **5g** (IC_50_ = 7.22 *µ*M) showed the highest hCA II inhibitory activity. Structure-activity relationships study showed that indolylchalcone derivatives have higher inhibitory activities than pyrido[2,3-d]pyrimidine derivatives on hCA I and hCA II. Additionally, methyl group bonded to uracil ring increases inhibitory activities on both hCA I and hCA II.

## 1. Introduction

Carbonic anhydrase (CA, EC 4.2.1.1) is a ubiquitous zinc enzyme. Basically, there are several mammalian cytosolic forms (CA-I, CA-II, CA-III, CA-VII, and CA-XIII), four membrane-bound forms (CA-IV, CA-IX, CA-XII, and CA-XIV), one mitochondrial form (CA-V), and a secreted CA form (CA-VI) [1, 2]. They all catalyze a very simple physiological reaction, the interconversion between carbon dioxide and the bicarbonate ion, and are thus involved in crucial physiological processes connected with respiration and transport of CO_2_/bicarbonate between metabolizing tissues and the lungs, pH and CO_2_ homeostasis, electrolyte secretion in a variety of tissues/organs, biosynthetic reactions (such as the gluconeogenesis, lipogenesis, and ureagenesis), bone resorption, calcification, tumorigenicity, and many other physiologic or pathologic processes [1–3]. CA inhibitors have now been a mainstay of human clinical intervention for several decades, with at least 25 clinically used drugs that are CA inhibitors [4]. Although there are many studies on this enzyme, the CA enzyme family continues to capture the attention of drug discovery scientists and clinicians as the knowledge regarding the therapeutic implications associated with this enzyme class continues to grow [4, 5].

Indoles are one of the most important nitrogen containing heterocyclic molecules, found extensively in biological system which play vital role in biochemical processes. Indole ring constitutes an important template for drug design such as the classical nonsteroidal anti-inflammatory drugs (NSAIDs) indomethacin and indoxole [6]. Further indole derivatives have been reported to possess promising biological activities including analgesic [7], antipyretic [8], antifungal [9], anti-inflammatory [10, 11], antitumor [12], anticonvulsant [13], and selective COX-2 inhibitory activities [14]. Thus the efficient synthesis of novel substituted indole derivative compounds still represents highly pursued target.

Pyrido[2,3-d]pyrimidines have received considerable attention over the past years because of their wide range of biological activities, which include antitumor [15], antibacterial [16], anti-inflammatory [17], and antifungal activities [18], and also act as cyclin-dependent kinase 4 inhibitors [19]. Also these compounds are considered to be important for synthetic drugs (e.g., barbituric acid derivatives), chemotherapeutic agents (e.g., sulfadiazine), and agricultural chemicals [20].

In this study, a series of 7 indolylchalcone and 11 new pyrido[2,3-d]pyrimidine derivatives containing indole ring were synthesized and their effects on human carbonic anhydrase (hCA) I and II were evaluated. Additionally, structure-activity relationship was examined.

## 2. Materials and Methods

### 2.1. General Chemistry

Melting points were taken on a Barnstead Electrothermal 9200. IR spectra were measured on a Shimadzu Prestige-21 (200 VCE) spectrometer. ^1^H and ^13^C NMR spectra were measured on a Varian Infinity Plus spectrometer at 300 and 75 Hz, respectively. ^1^H and ^13^C chemical shifts are referenced to the internal deuterated solvent. Mass spectra were obtained using MICROMASS Quattro LC-MS-MS spectrometer. Solvents were dried following standard methods. Sepharose 4B, L-tyrosine, sulfonamide, synthetic starting material, reagents, and solvents were purchased from Merck, Alfa Easer, Sigma-Aldrich, and Fluka.

### 2.2. Synthetic Procedures and Spectral Data

*1H-Indole-3-carbaldehyde (* ***2***). Phosphorous oxychloride (1 mL) was added dropwise to cold anhydrous DMF (3 mL) and the mixture was stirred at 0°C for 1 h. The indole (1.17 g), dissolved in anhydrous DMF, was added dropwise to above formylation complex solution at below 10°C. The mixture was warmed to 35–40°C and stirred for 1 hour. Then NaOH_(aq)_ (5.5 g NaOH, 14.6 mL water) was added. The mixture was warmed to 100°C and stirred for 1 h; then it was cooled, filtrated, and dried in vacuum oven for overnight. Pink powder was obtained in 94% yield. ^1^H NMR (CDCl_3_, 300 MHz) *δ*: 10.07 (1H, s), 8.82 (1H, s, NH), 8.32–8.34 (1H, dd,* J*_1_ = 2.0 Hz,* J*_2_ = 7.3 Hz), 7.85 (1H, d,* J* = 2.9 Hz), 7.44–7.46 (1H, m), 7.30–7.36 (2H, m) ppm; ^13^C NMR (CDCl_3_, 75 MHz) *δ*: 185.7, 139.2, 137.7, 124.8, 124.2, 122.8, 121.5, 118.8, 113.1 ppm.

*N-Methyl Indole-3-carboxaldehyde (* ***3***). K_2_CO_3_ (0.95 g) and CH_3_I (2 mL) were added to solution of indole-3-carboxaldehyde (1 g) in 10 mL DMF. The mixture was stirred at 100°C for 4 hours and then cooled and poured onto ice-water. The precipitate was filtrated and dried in vacuum oven. White solid was obtained in 95% yield. ^1^H NMR (CDCl_3_, 300 MHz) *δ*: 9.97 (1H, s), 8.29–8.31 (1H, dd,* J*_1_ = 1.8 Hz,* J*_2_= 7.3 Hz), 7.66 (1H, s), 7.29–7.37 (3H, m), 3.86 (3H, s) ppm; ^13^C NMR (CDCl_3_, 75 MHz) *δ*: 184.7, 139.9, 138.0, 125.3, 124.2, 123.1, 122.0, 118.0, 110.2, 33.8 ppm.

*Synthesis of Indolylchalcone Derivatives (* ***5a*** *–* ***g***). A solution of NaOH_(aq)_ (40%, 5 mL) was added to mixture of 1-methyl-1H-indole-3-carbaldehyde (1 mmol)** 3** and acetophenone derivatives (1 mmol)** 4a**–**g** in absolute ethanol. The mixture was stirred at room temperature for 2 hours. Then it was poured into ice-cold water, neutralized with acid, filtrated, and washed with water. The filtrate was dried in vacuum oven.

*(E)-1-(3,4-Dimethoxyphenyl)-3-(1-methyl-1H-indol-3-yl) prop-2-en-1-one (* ***5a***). Yield: 75%, yellow powder, mp: 271°C, IR (KBr): 3089.9, 3008.9, 2910.5, 2839.2, 1645.2, 1597.0, 1556.5, 1373.3, 1255.6, 1166.9, 1022.7, 804.3 *ν* (cm^−1^); ^1^H NMR (CDCl_3_, 300 MHz) *δ*: 8.08 (1H, d,* J* = 15.5 Hz), 8.01–8.03 (1H, dd,* J*_1_ = 2.0 Hz,* J*_2_ = 6.1 Hz), 7.71–7.74 (1H, dd,* J*_1_ = 1.8 Hz,* J*_2_ = 8.5 Hz), 7.66 (1H, d,* J* = 1.8 Hz), 7.57 (1H, d,* J* = 15.5 Hz), 7.47 (1H, s), 7.30–7.39 (3H, m), 6.95 (1H, d,* J* = 8.5 Hz), 3.99 (3H, s), 3.97 (3H, s), 3.84 (3H, s) ppm; ^13^C NMR (CDCl_3_, 75 MHz) *δ*: 189.1, 152.8, 149.2, 138.4, 138.1, 134.7, 132.3, 126.3, 123.3, 122.7, 121.7, 121.0, 116.6, 113.1, 110.8, 110.4, 110.1, 56.3, 56.2, 33.5 ppm; LC-MS (*m/z*): 322.57 [MH^+^].

*(E)-1-(4-Methoxyphenyl)-3-(1-methyl-1H-indol-3-yl) prop-2-en-1-one (* ***5b*** *). *Yield: 65%, yellow powder, mp: 254°C, IR (KBr): 3128.5, 3045.6, 2935.6, 2841.1, 1649.1, 1598.9, 1373.3, 1253.7, 1166.9, 1026.1 *ν* (cm^−1^); ^1^H NMR (CDCl_3_, 300 MHz) *δ*: 8.00–8.10 (4H, m), 7.57 (1H, d,* J* = 15.5 Hz), 7.46 (1H, s), 7.30–7.37 (3H, m), 7.00 (2H, d,* J* = 8.8 Hz), 3.89 (3H, s), 3.83 (3H, s) ppm; ^13^C NMR (CDCl_3_, 75 MHz) *δ*: 187.2, 161.2, 136.4, 136.1, 132.7, 130.1, 128.7, 124.3, 121.5, 119.7, 119.0, 114.9, 111.9, 111.2, 108.4, 53.7, 31.5 ppm; LC-MS (*m/z*): 293.00 [MH^+^].

*(E)-3-(1-Methyl-1H-indol-3-yl)-1-p-tolylprop-2-en-1-One (* ***5c***). Yield: 85%, dark yellow powder, mp: 240°C, IR (KBr): 3101.5, 3022.4, 2914.4, 1647.2, 1579.7, 1556.5, 1371.3, 1280.7, 1174.6, 1029.9 804.3 *ν* (cm^−1^); ^1^H NMR (CDCl_3_, 300 MHz) *δ*: 8.08 (1H, d,* J* = 15.5 Hz), 8.00–8.03 (1H, dd,* J*_1_ = 2.0 Hz,* J*_2_ = 6.5 Hz), 7.97 (2H, d,* J* = 8.0 Hz), 7.55 (1H, d,* J* = 15.5 Hz), 7.46 (1H, s), 7.31–7.39 (3H, m), 7.29 (2H, d,* J* = 8.0 Hz), 3.84 (3H, s), 2.44 (3H, s) ppm; ^13^C NMR (CDCl_3_, 75 MHz) *δ*: 190.4, 143.1, 138.5, 138.4, 136.7, 134.7, 129.4, 128.6, 126.3, 123.3, 121.7, 121.0, 117.1, 113.2, 110.4, 33.5, 21.9 ppm, LC-MS (*m/z*): 276.25 [MH^+^].

*(E)-1-(4-Chlorophenyl)-3-(1-methyl-1H-indol-3-yl)prop-2-en-1-one (* ***5d***). Yield: 96%, yellow powder, mp: 248°C, IR (KBr): 3103.4, 3085.2, 2908.8, 2807.7, 1645.2, 1580.7, 1371.9, 1282.6, 1029.9, 1008.7, *ν* (cm^−1^); ^1^H NMR (CDCl_3,_ 300 MHz) *δ*: 8.09 (1H, d,* J* = 15.5 Hz), 7.98–8.02 (3H, m), 7.46–7.52 (4H, m), 7.32–7.40 (3H, m), 3.85 (3H, s) ppm; ^13^C NMR (CDCl_3_, 75 MHz) *δ*: 189.6, 139.5, 138.7, 138.5, 137.6, 135.3, 129.9, 129.0, 126.3, 123.5, 121.9, 121.1, 116.4, 113.1, 110.5, 33.6 ppm; LC-MS (*m/z*): 296.61 [MH^+^].

*(E)-3-(1-Methyl-1H-indol-3-yl)-1-phenylprop-2-en-1-one (* ***5e***). Yield: 86%, light yellow powder, mp: 227°C, IR (KBr): 3095.7, 3055.2, 2933.7, 1643.3, 1581.6, 1554.6, 1462.0, 1371.3, 1278.8, 1213.2, 1076.2 *ν* (cm^−1^); ^1^H NMR (CDCl_3_, 300 MHz) *δ*: 8.09 (1H, d,* J* = 15.2 Hz), 8.00–8.06 (3H, m), 7.47–7.57 (5H, m), 7.29–7.39 (3H, m), 3.84 (3H, s) ppm; ^13^C NMR (CDCl_3_, 75 MHz) *δ*: 190.9, 139.3, 138.9, 138.4, 135.0, 132.4, 128.7, 128.5, 126.3, 123.4, 121.8, 121.0, 117.0, 113.1, 110.4, 33.5 ppm; LC-MS (*m/z*): 262.26 [MH^+^].

*(E)-3-(1-Methyl-1H-indol-3-yl)-1-(4-nitrophenyl)prop-2-en-1-one (* ***5f***). Yield: 88%, orange powder, mp: 278°C, IR (KBr): *ν* (cm^−1^); 3097.68, 3035.96, 1903.74, 1737.86, 1649.14, 1604.77, 1558.48, 1517.98, 1340.53, 1209.37, 813.96. ^1^H NMR (CDCl_3_, 300 MHz) *δ*: 8.32–8.39 (3H, m), 8.18 (1H, s), 8.13–8.16 (1H, m), 8.07 (1H, d,* J* = 15.5 Hz), 7.63 (1H, d,* J* = 15.5 Hz), 7.58 (1H, s), 7.29–7.35 (3H, m), 3.88 (3H, s) ppm; ^13^C NMR (CDCl_3_, 75 MHz) *δ*: 183.4, 140.0, 136.0, 134.4, 131.0, 125.1, 121.5, 120.6, 119.5, 118.5, 117.3, 116.2, 110.6, 108.3, 105.8, 29.0 ppm; LC-MS (*m/z*): 307.52 [MH^+^].

*(E)-3-(1-Methyl-1H-indol-3-yl)-1-(3-nitrophenyl)prop-2-en-1-one (* ***5g***). Yield: 87%, light orange powder, mp: 267°C, IR (KBr): 3099.6, 2605.8, 1649.1, 1583.5, 1519.9, 1467.8, 1369.4, 1342.4, 1282.6, 1070.4, 819.7 *ν* (cm^−1^); ^1^H NMR (CDCl_3_, 300 MHz) *δ*: 8.86 (1H, s), 8.39 (2H, t,* J* = 8.2 Hz), 8.17 (1H, d,* J* = 15.5 Hz), 7.99–8.02 (1H, m), 7.70 (1H, t,* J* = 7.9 Hz), 7.55 (1H, s), 7.50 (1H, d,* J* = 15.5 Hz), 7.33–7.43 (3H, m), 3.87 (3H, s) ppm; ^13^C NMR (CDCl_3_, 75 MHz) *δ*: 183.3, 143.7, 136.0, 133.8, 131.0, 129.4, 125.1, 121.9, 121.5, 118.9, 118.5, 117.5, 116.2, 110.6, 108.3, 105.8, 28.9 ppm; LC-MS (*m/z*): 307.29 [MH^+^].

*Synthesis of Pyrido[2,3-d]pyrimidines Derivatives *(***7a*** *–* ***k***). A mixture of 6-aminouracil derivatives (1 mmol)** 6a** or** 6b**, chalcone derivatives** 5a**–**g** (1 mmol), and NaOH (1 mmol) in 30 mL absolute ethanol was refluxed for 18 hours. The mixture was cooled and poured into ice-cold water. The precipitate was filtrated, washed with water, and dried in vacuum oven for overnight. The crude products were recrystallized from ethanol.

*7-(3,4-Dimethoxyphenyl)-1-methyl-5-(1-methyl-1H-indol-3-yl)pyrido[2,3-d]pyrimidine-2,4(1H,3H)-dione (* ***7a***). Yield: 90%, dark yellow powder, mp: 333-334°C, IR (KBr): 3169.3, 3044.5, 2940.1, 2835.6, 1685.9, 1583.8, 1518.4, 1387.6, 1256.7, 1023.8 *ν* (cm^−1^); ^1^H NMR (CDCl_3_, 300 MHz): 10.88 (1H, s, NH), 7.81–7.85 (2H, m), 7.75 (1H, s), 7.70 (1H, s), 7.51 (1H, d,* J* = 8.2 Hz), 7.40 (1H, d,* J* = 7.6 Hz), 7.20 (1H, t,* J* = 7.5 Hz), 7.03–7.12 (2H, m), 3.89 (3H, s), 3.87 (3H, s), 3.84 (3H, s), 3.66 (3H, s) ppm; ^13^C NMR (CDCl_3_, 75 MHz): *δ* 160.6, 158.3, 152.4, 151.5, 151.4, 149.4, 148.3, 136.9, 130.8, 130.5, 127.4, 122.3, 120.8, 120.7, 119.8, 118.4, 112.8, 111.2, 110.3, 110.1, 106.1, 56.2, 56.1, 33.4, 30.0 ppm; LC-MS (*m/z*): 443.41 [MH^+^].

*1-Methyl-5-(1-methyl-1H-indol-3-yl)-7-p-tolylpyrido[2,3-d]pyrimidine-2,4(1H,3H)-dione (* ***7b***). Yield: 55%, light yellow powder, mp: 399-400°C, IR (KBr): 3165.1, 3047.1, 2848.8, 1685.7, 1589.3, 1519.9, 1394.5, 1257.5, 1093.6 *ν* (cm^−1^); ^1^H NMR (CDCl_3_, 300 MHz) *δ*: 11.39 (1H, s, NH), 8.14 (2H, d,* J* = 8.2 Hz), 7.79 (1H, s), 7.71 (1H, s), 7.52 (1H, d,* J* = 7.6 Hz), 7.42 (1H, d,* J* = 7.6 Hz), 7.36 (2H, d,* J* = 8.2 Hz), 7.21 (1H, t,* J* = 7.3 Hz), 7.07 (1H, t,* J* = 7.3 Hz), 3.86 (3H, s), 3.66 (3H, s), 2.39 (3H, s) ppm; ^13^C NMR (CDCl_3_, 75 MHz): *δ* 161.0, 156.6, 154.1, 151.3, 150.7, 149.5, 139.6, 139.2, 133.5, 131.9, 129.9, 129.6, 125.7, 122.1, 120.5, 118.3, 113.2, 110.9, 107.8, 33.4, 29.4, 24.1 ppm; LC-MS (*m/z*): 396.98 [MH^+^].

*7-(4-Chlorophenyl)-1-methyl-5-(1-methyl-1H-indol-3-yl)pyrido[2,3-d]pyrimidine-2,4(1H,3H)-dione (* ***7c***). Yield: 60%, light yellow powder, mp: 385–389°C, IR (KBr): 3172.9, 3043.6, 2927.9, 2848.8, 1685.9, 1521.0, 1392.6, 1257.9, 1091.7, 1012.8, 729.0 *ν* (cm^−1^); ^1^H NMR (DMSO, 300 MHz) *δ*: 11.40 (1H, s, NH), 8.26 (2H, d,* J* = 8.5 Hz), 7.78 (1H, s), 7.74 (1H, s), 7.59 (2H, d,* J* = 8.5 Hz), 7.49 (1H, d,* J* = 8.2 Hz), 7.40 (1H, d,* J* = 7.3 Hz), 7.18 (1H, t,* J* = 7.6 Hz), 7.04 (1H, t,* J* = 7.6 Hz), 3.86 (3H, s), 3.62 (3H, s) ppm; ^13^C NMR (CDCl_3_, 75 MHz) *δ*: 161.0, 156.6, 154.1, 151.3, 148.2, 137.1, 136.6, 135.9, 131.9, 129.7, 129.6, 127.2, 122.1, 120.6, 120.5, 118.3, 113.2, 110.9, 107.8, 33.4, 29.5 ppm; LC-MS (*m/z*): 418.36 [MH^+^].

*1-Methyl-5-(1-methyl-1H-indol-3-yl)-7-(3-nitrophenyl)pyrido[2,3-d]pyrimidine-2,4(1H,3H)-dione (* ***7d***). Yield: 41%, mustard powder, mp: 397°C, IR (KBr): 3176.7, 3049.4, 1681.9, 1591.2, 1523.7, 1454.3, 1346.3, 1253.7, 1085.9, 732.9 *ν* (cm^−1^); ^1^H NMR (CDCl_3_, 300 MHz) *δ*: 12.01 (1H, s, NH), 8.98 (1H, t,* J* = 1.7 Hz), 8.66 (1H, d,* J* = 8.2 Hz), 8.32–8.35 (1H, dd,* J*_1_ = 1.5 Hz,* J*_2_ = 8.2 Hz), 7.82 (1H, d,* J* = 8.2 Hz), 7.79 (2H, s), 7.50 (1H, d,* J* = 8.2 Hz), 7.42 (1H, d,* J* = 7.6 Hz), 7.20 (1H, t,* J* = 7.0 Hz), 7.05 (1H, t,* J* = 7.0 Hz), 3.88 (3H, s), 3.65 (3H, s) ppm; ^13^C NMR (CDCl_3_, 75 MHz) *δ*: 161.2, 157.3, 152.8, 152.6, 150.0, 149.8, 138.8, 138.4, 137.5, 134.0, 131.9, 130.9, 127.2, 125.5, 120.8, 120.7, 119.6, 118.3, 113.3, 110.9, 108.9, 33.5, 28.9 ppm; LC-MS (*m/z*): 428.66 [MH^+^].

*7-(3,4-Dimethoxyphenyl)-1,3-dimethyl-5-(1-methyl-1H-indol-3-yl)pyrido[2,3-d]pyrimidine-2,4(1H,3H)-dione (* ***7e***). Yield: 50%, yellow powder, mp: 340-341°C, IR (KBr): 3078.3, 2937.9, 2839.2, 1693.5, 1647.1, 1419.6, 1329.4, 1220.9, 1134.1, 1022.2 *ν* (cm^−1^); ^1^H NMR (CDCl_3_, 300 MHz): 7.70–7.75 (3H, m), 7.54 (1H, d,* J* = 8.2 Hz), 7.51 (1H, s), 7.41 (1H, d,* J* = 8.2 Hz), 7.31 (1H, d,* J* = 7.0 Hz), 7.18 (1H, t,* J* = 7.0 Hz), 6.98 (1H, d,* J* = 8.2 Hz), 3.99 (3H, s), 3.96 (3H, s), 3.91 (3H, s), 3.90 (3H, s), 3.42 (3H, s) ppm; ^13^C NMR (CDCl_3_, 75 MHz): 161.0, 158.3, 152.5, 151.8, 151.4, 149.4, 148.3, 136.9, 130.8, 130.5, 127.4, 122.3, 120.8, 120.7, 119.8, 118.4, 112.8, 111.2, 110.3, 110.1, 106.1, 56.2, 56.1, 33.4, 30.3, 28.7 ppm; LC-MS (*m/z*): 458.18 [MH^+^].

*7-(4-Methoxyphenyl)-1,3-dimethyl-5-(1-methyl-1H-indol-3-yl)pyrido[2,3-d]pyrimidine-2,4(1H,3H)-dione (* ***7f***). Yield: 20%, yellow powder, mp: 335.7–336°C, IR (KBr): 3057.1, 2960.7, 2839.2, 1699.2, 1651.0, 1516.0, 1473.6, 1356.6, 1024.2 *ν* (cm^−1^); ^1^H NMR (CDCl_3_, 300 MHz): 8.11 (2H, d,* J* = 8.8 Hz), 7.69 (1H, s), 7.54 (1H, d,* J* = 7.8 Hz), 7.50 (1H, s), 7.40 (1H, d,* J* = 8.2 Hz), 7.27(1H, d,* J* = 7.0 Hz), 7.17 (1H, t,* J* = 7.8 Hz), 7.01 (2H, d,* J* = 8.8 Hz), 3.90 (3H, s), 3.88 (6H, s), 3.42 (3H, s) ppm; ^13^C NMR (CDCl_3_, 75 MHz): 161.6, 160.8, 158.1, 152.3, 151.6, 148.0, 136.7, 130.6, 130.0, 128.9, 127.2, 122.0, 120.4, 119.6, 118.0, 114.2, 112.6, 109.8, 105.8, 55.4, 33.2, 30.1, 28.4 ppm; LC-MS (*m/z*): 428.04 [MH^+^].

*1,3-Dimethyl-5-(1-methyl-1H-indol-3-yl)-7-p-tolylpyrido[2,3-d]pyrimidine-2,4(1H,3H)-dione (* ***7g***). Yield: 40%, yellow powder, mp: 340-341°C, IR (KBr): 3021.3, 2960.7, 2908.6, 1707.0, 1660.7, 1523.7, 1473.6, 1394.5, 1365.6, 1257.5, 1016.4 *ν* (cm^−1^); ^1^H NMR (CDCl_3_, 300 MHz) *δ*: 8.04 (2H, d,* J* = 8.2 Hz), 7.73 (1H, s), 7.54 (1H, d,* J* = 8.2 Hz), 7.51 (1H, s), 7.39 (1H, d,* J* = 8.2 Hz), 7.25–7.32 (3H, m), 7.17 (1H, t,* J* = 7.3 Hz), 3.90 (3H, s), 3.89 (3H, s), 3.42 (3H, s), 2.43 (3H, s) ppm; ^13^C NMR (CDCl_3_, 75 MHz) *δ*: 160.9, 158.6, 152.4, 151.6, 148.2, 140.8, 136.7, 134.8, 130.6, 129.6, 127.3, 127.2, 122.1, 120.5, 119.6, 118.5, 112.6, 109.9, 106.2, 33.2, 30.2, 28.4, 21.4 ppm; LC-MS (*m/z*): 411.33 [MH^+^].

*7-(4-Chlorophenyl)-1,3-dimethyl-5-(1-methyl-1H-indol-3-yl)pyrido[2,3-d]pyrimidine 2,4(1H,3H)-dione (* ***7h***). Yield: 76%, yellow powder, mp: 358-359°C, IR (KBr): 3064, 3045, 2943, 1701, 1654 *ν* (cm^−1^); ^1^H NMR (CDCl_3_, 300 MHz) *δ*: 8.07 (2H, d,* J* = 8.5 Hz), 7.73 (1H, s), 7.52–7.55 (2H, m), 7.47 (2H, d,* J* = 8.5 Hz), 7.41 (1H, d,* J* = 8.2 Hz), 7.29 (1H, t,* J* = 7.5 Hz), 7.18 (1H, t,* J* = 7.0 Hz), 3.90 (3H, s), 3.88 (3H, s), 3.42 (3H, s) ppm; ^13^C NMR (CDCl_3_, 75 MHz): *δ* 161.0, 157.5, 152.7, 151.8, 148.8, 136.9, 136.8, 136.2, 131.0, 129.3, 128.9, 127.3, 122.4, 120.8, 119.8, 118.8, 112.6, 110.2, 106.8, 33.5, 30.5, 28.7 ppm; LC-MS (*m/z*): 432.37 [MH^+^].

*1,3-Dimethyl-5-(1-methyl-1H-indol-3-yl)-7-phenylpyrido[2,3-d]pyrimidine-2,4(1H,3H)-dione (* ***7i***). Yield: 32%, yellow powder, mp: 320°C, IR (KBr): 3070.6, 3037.8, 2933.7, 1697.3, 1651.0, 1591.2, 1533.4, 1419.6, 1390.6, 1259.5, 1053.1 *ν* (cm^−1^); ^1^H NMR (CDCl_3_, 300 MHz) *δ*: 8.13–8.15 (2H, dd,* J*_1_ = 2.0 Hz,* J*_2_ = 7.5 Hz), 7.77 (1H, s), 7.49–7.56 (5H, m), 7.41 (1H, d,* J* = 8.2 Hz), 7.29 (1H, t,* J* = 7.0 Hz), 7.18 (1H, t,* J* = 7.0 Hz), 3.91 (3H, s), 3.90 (3H, s), 3.42 (3H, s) ppm; ^13^C NMR (CDCl_3_, 75 MHz) *δ*: 161.1, 158.7, 152.6, 151.8, 148.6, 137.8, 136.9, 131.0, 130.6, 129.1, 127.6, 127.4, 122.4, 120.7, 119.9, 119.1, 112.7, 110.1, 106.6, 33.5, 30.5, 28.7 ppm; LC-MS (*m/z*): 397.51 [MH^+^].

*1,3-Dimethyl-5-(1-methyl-1H-indol-3-yl)-7-(4-nitrophenyl)pyrido[2,3-d]pyrimidine-2,4(1H,3H)-dione (* ***7j***). Yield: 60%, light orange powder, mp: 347–349°C, IR (KBr): 3157.4, 3049.4, 2945.3, 1703.1, 1658.7, 1585.4, 1519.9, 1342.4, 852.5 *ν* (cm^−1^); ^1^H NMR (CDCl_3_, 300 MHz) *δ*: 8.52 (2H, d,* J* = 7.9 Hz), 8.37 (2H, d,* J* = 7.9 Hz), 7.92 (1H, s), 7.82 (1H, s), 7.53 (1H, d,* J* = 8.2 Hz), 7.42 (1H, d,* J* = 7.9 Hz), 7.23 (1H, t,* J* = 7.1 Hz), 7.08 (1H, t,* J* = 7.0 Hz), 3.90 (3H, s), 3.75 (3H, s), 3.24 (3H, s) ppm; ^13^C NMR (CDCl_3_, 75 MHz) *δ*: 160.8, 155.3, 152.8, 151.6, 148.9, 148.7, 137.2, 134.4, 132.0, 129.2, 127.2, 124.6, 121.7, 120.7, 119.8, 118.6, 113.3, 110.9, 108.1, 33.4, 30.5, 28.8 ppm; LC-MS (*m/z*): 442.19 [MH^+^].

1*,3-Dimethyl-5-(1-methyl-1H-indol-3-yl)-7-(3-nitrophenyl)pyrido[2,3-d]pyrimidine-2,4(1H,3H)-dione (* ***7k***). Yield: 70%, mustard powder, mp: 349-350°C, IR (KBr): 3344.5, 3170.9, 3074.5, 2929.8, 1703.1, 1656.8, 1591.2, 1477.4, 1352.1, 1031.9 *ν* (cm^−1^); ^1^H NMR (CDCl_3_, 300 MHz) *δ*: 8.99 (1H, s), 8.69 (1H, d,* J* = 8.0 Hz), 8.34 (1H, d,* J* = 8.0 Hz), 7.92 (1H, s), 7.80–7.86 (2H, m), 7.52 (1H, d,* J* = 8.2 Hz), 7.39 (1H, d,* J* = 6.9 Hz), 7.20 (1H, t,* J* = 7.0 Hz), 7.07 (1H, t,* J* = 7.0 Hz), 3.89 (3H, s), 3.74 (3H, s), 3.21 (3H, s) ppm; ^13^C NMR (CDCl_3_, 75 MHz) *δ*: 160.3, 157.4, 153.3, 152.8, 150.0, 149.9, 138.4, 138.2, 137.2, 134.0, 132.0, 130.9, 127.2, 125.8, 120.7, 120.5, 119.8, 118.6, 113.2, 110.9, 108.7, 33.5, 30.6, 28.9 ppm; LC-MS (*m/z*): 442.17 [MH^+^].

### 2.3. Preparation and Purification of Hemolysate from Blood Red Cells

Blood samples (25 mL) were taken from healthy human volunteers. They were anticoagulated with acid-citrate-dextrose and centrifuged at 2000 g for 20 min at 4°C and the supernatant was removed. The packed erythrocytes were washed three times with 0.9% NaCl and then haemolysed in cold water. The ghosts and any intact cells were removed by centrifugation at 2000 g for 25 min at 4°C, and the pH of the haemolysate was adjusted to pH 8.5 with solid Tris-base. The 25 mL haemolysate was applied to an affinity column containing L-tyrosine-sulfonamide-sepharose-4B [21] equilibrated with 25 mM Tris-HCl/0.1 M Na_2_SO_4_ (pH 8.5). The affinity gel was washed with 50 mL of 25 mM Tris-HCl/22 mM Na_2_SO_4_ (pH 8.5). The hCA isozymes were then eluted with 0.1 M NaCl/25 mM Na_2_HPO_4_ (pH 6.3) and 0.1 M CH_3_COONa/0.5 M NaClO_4_ (pH 5.6), which recovered hCA I and hCA II, respectively. Fractions of 3 mL were collected and their absorbance was measured at 280 nm.

### 2.4. CA Enzyme Assay

CA activity was measured by the Maren method which is based on determination of the time required for the pH to decrease from 10.0 to 7.4 due to CO_2_ hydration [22]. The assay solution was 0.5 M Na_2_CO_3_/0.1 M NaHCO_3_ (pH 10.0) and Phenol Red was added as the pH indicator. CO_2_-hydratase activity (enzyme units (EU)) was calculated by using the equation *t*_0_ − *t*_*c*_/*t*_*c*_, where *t*_0_ and *t*_*c*_ are the times for pH change of the nonenzymatic and the enzymatic reactions, respectively.

### 2.5. In Vitro Inhibition Studies

For the inhibition studies of indolylchalcone and pyrido[2,3-d]pyrimidine derivatives, different concentrations of these compounds were added to the enzyme. Activity percentage values of CA for different concentrations of each pyrimidine derivatives were determined by regression analysis using Microsoft Office 2000 Excel. CA enzyme activity without these compounds was accepted as 100% activity.

## 3. Results and Discussion

### 3.1. Chemistry

**Scheme 1:**
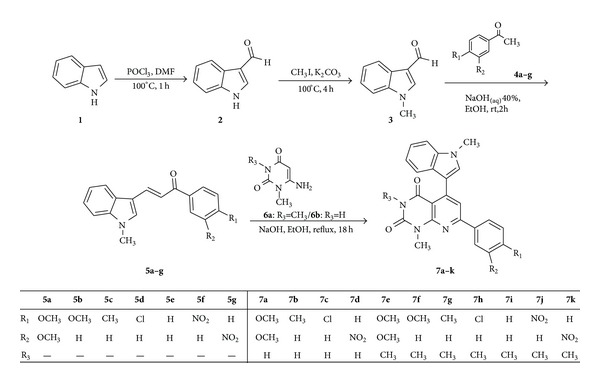
Synthesis of pyrido[2,3-d]pyrimidine derivatives (**7a**–**k**).

The synthetic procedures are depicted in Scheme 1. The indolylchalcone derivatives** 5a**–**g**, prepared by the condensing various acetophenones and indolylaldehyde** 3** with NaOH as a base, were reacted with 3-methyl-6-aminouracil** 6a** and 6-aminouracil** 6b** to get pyrido[2,3-d]pyrimidine derivatives (**7a**–**k**) at high yields. The large* J* value (15.5 Hz) clearly reveals the* E*-geometry for the chalcones.

### 3.2. Biological Evaluation of Indolylchalcone and Pyrido[2,3-d]pyrimidine Derivatives for hCA I and hCA II Inhibitory Activities

For evaluating the hCA I and II inhibitory effect, all compounds were subjected to hCA I and II inhibition assay with CO_2_ as a substrate. The result showed that all synthesized compounds (**5a**–**g** and** 7a**–**k**) inhibited the hCA I and hCA II enzyme activities.

**Figure 1:**
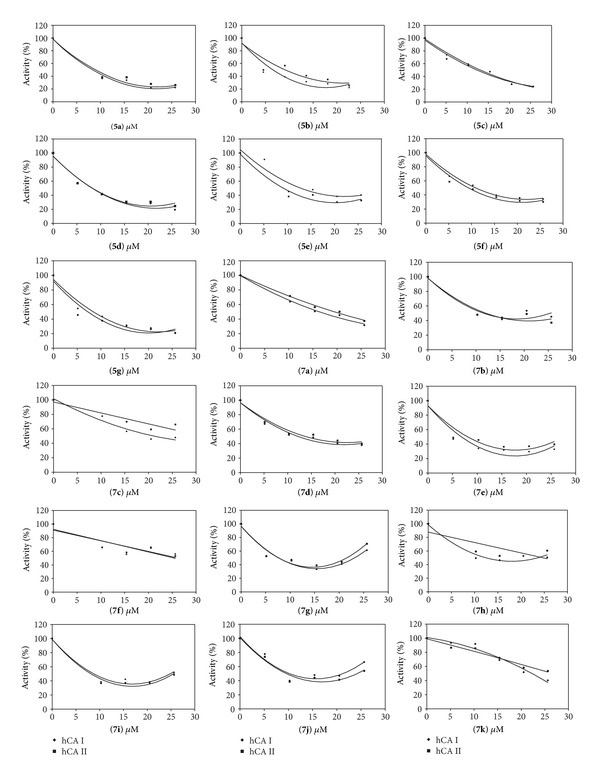
IC_50_ graphics of indolylchalcone (**5a**–**g**) and pyrido[2,3-d]pyrimidine (**7a**–**k**) derivatives on hCA I and hCA II.

The IC_50_ values and inhibition constants of** 5a**–**g** and** 7a**–**k** analogues against hCA I and hCA II were summarized in Table 1 and the IC_50_ graphs were given in Figure 1.

We have determined the IC_50_ values of 6.79–26.21 *μ*M for the inhibition of hCA I and 7.22–31.10 *μ*M for the inhibition of hCA II. Among all compounds,** 7e** (IC_50_ = 6.79 *μ*M) was found to be the most active one for hCA I inhibitory activity and** 5g** (IC_50_ = 7.22 *μ*M) showed the highest hCA II inhibitory activity.** 5b** (IC_50_ = 7.42*μ*M) was found to be the most active one for hCA I inhibitory activity and** 5g** (IC_50_ = 7.22 *μ*M) showed the highest hCA II inhibitory activity for the indolylchalcone derivatives. Among the pyrido[2,3-d]pyrimidine derivatives,** 7e** (IC_50_ = 6.79 *μ*M) showed the highest hCA I inhibitory activity and** 7g** (IC_50_ = 7.57 *μ*M) showed the highest hCA II inhibitory activity.

It was reported that 1,4-dihydropyrimidinone substituted diarylurea compounds were synthesized and their effects on the hCA I and II enzyme activities were examined. Their minimum concentrations to achieve 50% inhibition were between 66.23 and 197.70 *μ*M for hCA I, 63.09 and 169.71 *μ*M for hCA II [23]. It is evident that the indolylchalcone and pyrido[2,3-d]pyrimidine derivatives, synthesized in this work, showed better hCA I and II inhibitory activities than 1,4-dihydropyrimidinone substituted diarylurea compounds.

### 3.3. Structure-Activity Relationships (SAR)

Generally, we have seen that indolylchalcone derivatives have higher inhibitory activities than pyrido[2,3-d]pyrimidine derivatives on hCA I and hCA II. The following structure-activity relationship (SAR) observations can be drawn from the data.

- For the indolylchalcone derivatives, the presence of one electron-donating group (methoxy) bonded to paraposition of phenyl ring (**5b**) increased inhibitory activity on hCA I. Electron-withdrawing group (nitro) bonded to metaposition of phenyl ring (**5g**) has the highest hCA II inhibitory activity (IC_50_ = 7.22 *μ*M).
- For the pyrido[2,3-d]pyrimidine derivatives, (i) the compounds (**7e**,** 7g**, and** 7h**) which have methyl group at the 3-position of uracil ring showed a higher inhibitory effect than the compounds (**7a**–**c**) which have hydrogen atom at the 3-position of uracil ring and have the same groups at the phenyl ring against both hCA I and hCA II (compare** 7e** with** 7a**,** 7g** with** 7b**, and** 7h** with** 7c**). (ii) Electron-withdrawing group (nitro) bonded to metaposition of phenyl ring (**7k**) has a very low hCA I inhibitory activity (IC_50_ = 22.30 *μ*M). (iii) Mostly, the pyrido[2,3-d]pyrimidine derivatives have higher inhibitory activities on hCA I than hCA II.

Sulfonamides are coordinated to the zinc (II) ion within the hCA active site, whereas their organic scaffold fills the entire active site cavity, making an extensive series of van der Waals and polar interactions with amino acid residues delimiting this cavity [24, 25]. As the synthesized compounds are very bulky and do not contain a classical zinc-binding group [4], it can be hypothesized that they are not able to bind near the zinc ion showing a different mechanism of action. Structural studies of the complexes that these compounds form with the human isoform II could clarify this important issue.

## 4. Conclusions

In conclusion, series of 7 indolylchalcone and 11 new pyrido[2,3-d]pyrimidine derivatives containing indole ring were synthesized. Their activities as hCA I and hCA II inhibitors and structure-activity relationships were examined. All compounds inhibited both hCA I and hCA II enzyme activities. Most of compounds containing electron-donating groups at phenyl ring were generally stronger inhibitors of hCA I and hCA II. Additionally, methyl group bonded to 3-position of uracil ring generally increased inhibitory activities on both hCA I and hCA II. Thus, the present study revealed that the type and position of substituent of the phenyl and uracil rings could be exploited to modulate the CA inhibitors efficacy.

In summary, enzyme inhibition is an important issue for drug design [26–28]. Our results showed that new pyrido[2,3-d]pyrimidine derivatives inhibited the hCA I and II enzyme activity. Therefore, the compounds here investigated are likely to be adopted as good candidates as drugs and may be taken for further evaluation in in vivo studies.

## Figures and Tables

**Table 1:** Inhibitory effect of indolylchalcone (**5a–g**) and pyrido[2,3-d]pyrimidine derivatives (**7a–k**) on hCA I and hCA II.

Compound	hCA I IC_50_ (*μ*M)	hCA II IC_50_ (*μ*M)
**5a**	8.34	8.88
**5b**	7.42	10.35
**5c**	13.07	12.28
**5d**	8.20	8.26
**5e**	12.84	9.15
**5f**	10.87	9.31
**5g**	8.38	7.22
**7a**	16.29	19.42
**7b**	11.56	12.06
**7c**	21.09	31.10
**7d**	12.14	13.66
**7e**	6.79	8.06
**7f**	26.21	25.40
**7g**	7.61	7.57
**7h**	12.36	24.67
**7i**	8.72	8.14
**7j**	10.19	9.56
**7k**	22.30	26.68
